# Time for change at PROJ in 2021

**DOI:** 10.1186/s12969-021-00560-y

**Published:** 2021-05-01

**Authors:** Charles H. Spencer, Alberto Martini

**Affiliations:** 1 University of Mississippi, Jackson, MS, USA; 2 University of Genova, Genoa, Italy

We wish to thank the Pediatric Rheumatology European Society (PReS) for its ongoing sponsorship of our journal. PReS has supported our young journal in so many ways. It has agreed with our objective of aiding in the development of the small but rapidly expanding field of international pediatric rheumatology (PR). As far as having its own journal, our subspecialty, the field of PR, had several potential directions to go in the 2000’s and even now. Our journal, backed by the support of 80% of the pediatric rheumatologists who replied to a survey in 2002, chose to be an Open Access journal. The sponsorship of *Pediatric Rheumatology* (PROJ) by PReS in 2011 helped us maintain an Open Access journal in the last 10 years free for anyone to read in any part of the world. Any physician or clinician or parent with access to a computer and a search engine can read PROJ without having to pay for access to print journals. We cannot measure the effect of an open access journal for our subspecialty but we think it has been positive. Our Impact Factor has risen to a decent 2.5–2.6 level over the past 10 years and likely reflects much PR interest and support. But some change is coming now in how to pay for our article processing charges (APC), particularly as the number of papers financially supported by PReS has been decreasing every year.

Each author may apply for a discount from BioMed Central/Springer. Written proof from a business manager or administrator that the authors have no access to funds for the APC will allow a full discount for the APC in many cases. Authors whose institution is a member of BioMed Central may get a large discount. As always, authors from countries with limited resources may receive a substantial or full discount. Manuscripts from national, international and global PR organizations with funding challenges will also be eligible for major discounts. Some pediatric rheumatologists have grants that will help with journal costs. Many institutions and governments, especially in Europe and the United Kingdom, support publishing in Open Access journals and pay the APC.

So it is time in 2021 for PROJ to leave the nest of PReS and fly on its own financially. It is a developmental step and an inevitable one. It was planned in the beginning of PReS sponsorship that change was eventually coming. So we will do everything we can as Editors to provide help for APC funding for every author who needs it. Please refer to the journal’s website for information on the support available and how to get in touch if you have any questions.

## Abbreviations

PReS: Pediatric Rheumatology European Society
PROJ: Pediatric rheumatology online journal
PR: Pediatric rheumatology
APC: Article processing charge

